# Labor Complicated with Monstrosity

**Published:** 1854-11

**Authors:** J. Q. Egelston

**Affiliations:** Primrose, Iowa


### LABOR COMPLICATED WITH MONSTROSITY.

BY DR. J. Q. EGELSTON, PRIMROSE, IOWA.

Dec. 29th, 185–, was called in attendance upon Mrs. K., during her third confinement; found her general health good; the state of gestation had not been attended with any unusual symptoms, with the exception of a slight sanguineous discharge from the vagina, five or six weeks before her accouchment, and was at the time attributed to too great bodily exertion; since then she had worn a puerperal bandage; was informed that the "waters" had been discharged before my arrival at six o'clock, A. M.; the pains were light and unsteady, and soon altogether subsided until near twelve o'clock, M., when they returned and successively increased in frequency and severity until the labor terminated at 2 o'clock, P. M.

1st Examination. Perineum relaxed; vagina moist, also relaxed, soft, and cool; the os uteri dilating kindly, the presenting point soft; occupying the mouth of the womb, and much resembling the "bay of waters," though rather smooth to the touch; no distinguishable part of the fœtus could be felt; concluded that the "membranes" were still unruptured.

2nd Examination. An hour later, some progress had been made toward delivery; the presenting point, a soft round tumor, occupying the superior strait, in the side of which could be distinctly traced a fissure of an inch and a half or two inches in length, by an inch in depth; could not yet determine the part presenting.

3rd Examination. Twenty minutes later, the tumor entirely expelled, but still attached by its internal surface; the nature of the case was now made fully evident to have been a *liver* presentation by actual examination; the hands and feet were next following, and after these, the head and breach; the fœtus being doubled in the straits of the pelvis,or rather between them; and presenting at the inferior, where they, for a few moments, resisted all further expulsive efforts of the uterus, until a retrocession of the breach could be effected, and restrained until the next accession of uterine action, when the fœtus, placenta and membranes all came away together, without injury to the mother.

The after treatment prescribed was the same as is usual, in normal labors; her recovery was speedy, and without any untoward symptoms attending it. The fœtus had been dead for some time, and was much deformed; the head had the appearance of having been stretched at the centre of its long diameter, rendering its short one much less than symmetrical; there was emphysema of the scalp at one or two points; the nose was turned close to one side; and the face otherwise considerably distorted; the sternum lay in a diagonal position with the median line of the body; the ribs crooked, misplaced, and over the region of the heart were altogether wanting; here the integuments assumed all of the characteristic appearances of the pericardium, beneath which the heart could distinctly be felt; the liver and a part of the alimentary tube were situated entirely outside of the abdominal cavity, and were attached to its parietes immediately underneath the lower point of the breast bone; the body of the fœtus was twisted upon its axis at near the centre of the spinal column, to such an extent as to bring the posterior parts above this point, upon a parallel with the anterior, but below it; the hands were broad, flat, and short, and apparently without joints at the articulations of the wrist or fingers; the feet were similar in appearance, and joined to the legs at their internal lateral surface, also without joint; the funis formed the border of a thin membraneous attachment between the fœtus and itself, which could be traced as arising from the umbilicus upward, under the edge of the ribs, across the sternum neck and face, and finding an insertion along the whole length of the umbilical chord; no sexual organs were perceptible, and its whole bulk was fully equal to that of a well-formed, healthy seven month's child.

Such is an imperfect and hasty description of this mal-formation, and of the peculiar symptoms attending the progress of labor.

